# Therapeutic Dilemmas Regarding Anticoagulation, Pulmonary Embolism, and Diffuse Alveolar Hemorrhage Due to Behçet’s Disease

**DOI:** 10.7759/cureus.14429

**Published:** 2021-04-12

**Authors:** Obaidullah Ahmadzai, Engin Ozakin, Mustafa Emin Canakci, Nurdan Acar, Cengiz Korkmaz

**Affiliations:** 1 Emergency Medicine, Eskişehir Osmangazi University, Eskişehir, TUR; 2 Division of Rheumatology, Eskişehir Osmangazi University, Eskişehir, TUR

**Keywords:** alveolar hemorrhage, pulmonary thromboembolism, behçet’s disease

## Abstract

The use of anticoagulants is still a matter of debate in deep venous thrombosis (DVT) and other thrombotic events in Behcet's disease (BD). Anticoagulant therapy is an integral part of treatment in cases of a pulmonary embolism (PE) that develops in other disorders. The issue of how to act when a pulmonary artery thrombosis is reported in the Behçet's patient may pose a major dilemma among emergency physicians. A 61-year-old male came to our ED with a complaint of chest pain and hemoptysis. The patient had tachypnea, dyspnea, tachycardia, a decrease of breath sounds in the basal regions of both lungs, and a few crackling rales were heard in the left lung field. Chest CT angiography showed pulmonary thromboembolism in the right middle and lower lobe segment arteries with pulmonary infarction as well as ground glass densities compatible with alveolar hemorrhage. High-dose steroid and cyclophosphamide were administered immediately without anticoagulant therapy based on pulmonary vasculitis and de novo clot formation in the pulmonary circulation. Clinical improvement was observed after four days of admission. The patient remained under observation with oral prednisolone and cyclophosphamide monthly. PE is almost non-existent in patients with BD, and signs of pulmonary artery thrombosis are associated with pulmonary vasculitis. Delaying immunosuppressive therapy may result in unwanted results in these kinds of patients. This case underlines the importance of recognizing this manifestation early to prevent potentially fatal consequences.

## Introduction

Behçet’s disease (BD) is a vasculitic disorder characterized by recurrent mucocutaneous, ocular, musculoskeletal, gastrointestinal, central nervous system, and vascular manifestations [1]. Pulmonary artery aneurysm is usually seen in young male BD patients and involvement is the main cause of mortality and morbidity [2]. However, pulmonary embolism (PE) involving the smaller arteries without pulmonary artery aneurysm is another type of involvement. As is well known that anticoagulant therapy is an integral part of treatment in cases of a PE that develops in other disorders. However, questioning the use of anticoagulants in BD patients with PE can sometimes lead to a delay in immunosuppressive therapy. This case report will likely increase awareness among ED physicians and prevents unnecessary anticoagulant use in these kinds of patients.

## Case presentation

**Figure 1:**
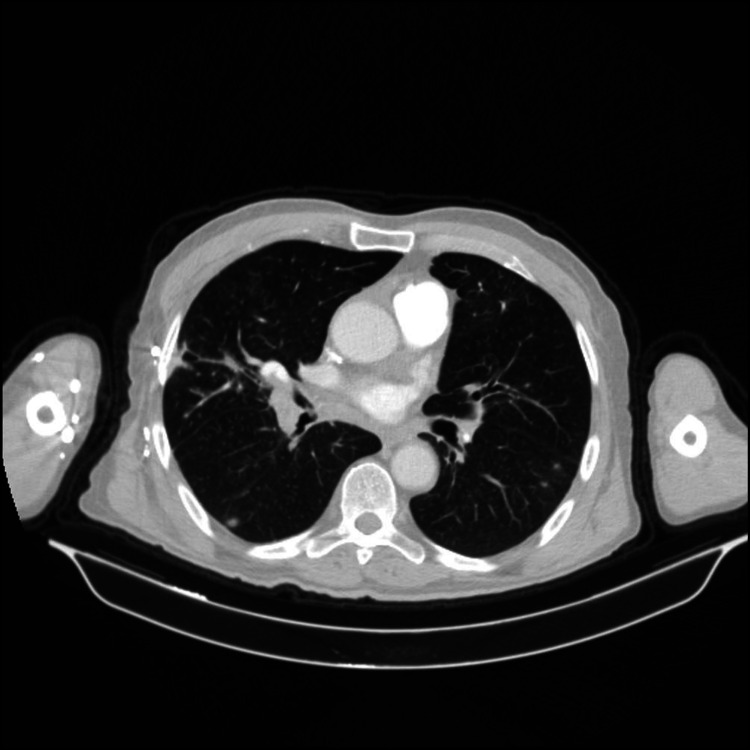
Right pulmonary embolism.

**Figure 2:**
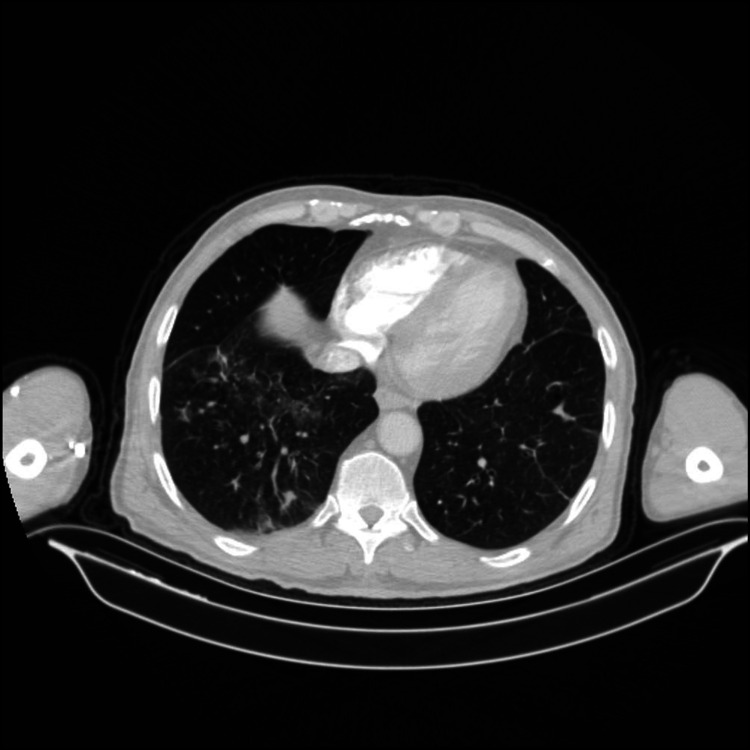
Alveolar hemorrhages.

A 61-year-old male presented to our ED with a complaint of chest pain and hemoptysis. His medical history consisted of BD for 22 years, diabetes mellitus for 9 years, and AA-type amyloidosis with nephrotic-range proteinuria for two years. AA type amyloidosis was considered due to BD. He was taking colchicine (1.5 mg per day), methylprednisolone (4 mg per day), and metformin 2000 mg per day. He developed dyspnea, cough, pleuritic chest pain, and hemoptysis three hours prior to presentation. At the ED, vital signs were normal. On auscultation of lungs, breath sounds decreased in the basal regions of both lungs, and few crackling rales were heard in the left lung field. The laboratory data were as follows: leukocyte count: 5800 per microliter of blood; hemoglobin: 12.4 g/dl; erythrocyte sedimentation rate (ESR): 80 mm/h; C-reactive protein (C-RP): 33.1 mg/L (normal range: 0-5 mg/L); fibrinogen: 718.1 mg/dl (normal range: 170-420 mg/L); D-dimer: 1.01 mcg/ml (normal range: 0-0.5 mg/L). Urea, creatinine levels, liver function tests, and electro­lytes were within normal limits. Urine analysis showed 3+ protein. The transthoracic cardiac echocardiography showed equalization in the measurement of the dimensions of the right and left ventricles. Chest CT angiography showed filling defects in the right middle and lower lobe segment arteries and hypodense lesion in the right lung superior segment consistent with pulmonary infarction and in the lower lobe of right lung ground glass densities compatible with alveolar hemorrhage (Figures 1 and 2). Lower extremity Doppler ultrasonography (USG) was normal. The patient was transferred to the rheumatology clinic for further evaluation. In the rheumatology clinic, the patient was considered as having PE due to BD-related pulmonary vasculitis; cyclophosphamide 1 g per month and pulse methylprednisolone (1 g/ 3 days) was started immediately. On the 4th day of the treatment, hemoptysis stopped and shortness of breath decreased. Doppler USG for upper extremities showed re-canalized partial thrombus starting from the right axillary vein and extending to the proximal of the brachial vein. The patient was discharged with methylprednisolone in a dose of 1 mg/kg, colchicine 1.5 mg per day, and metformin 2000 mg per day. At the follow-up visit one month later, the patient was symptom-free and the ESR and C-RP levels were 10 mm/h and 3 mg/L, respectively.

## Discussion

Herein, we report a case with BD who developed PE. The patient was successfully treated with immunosuppressive treatment without using an anticoagulant. When encountering such a patient, the first question to be asked is if thrombosis is due to BD or if the thromboembolic condition independent of BD? Making a definitive diagnosis is very important for the outcome of the disease. BD can lead to pulmonary artery aneurism (PAA) characterized by hemoptysis. It is the most important factor determining the outcome of the disease [3]. However, less frequently, PE is seen without PAA. Accidentally evaluating this situation as PE and using anticoagulant therapy only may lead to mortal results. In our patient, some distracting factors suggested the possibility of PE. It is well known that PAA and PE in the course of BD are seen in the first years of the disease and young male patients [2]. However, our patient was not young and the disease duration was quite long. This situation may suggest that PE may be caused by non-BD diseases. Moreover, our patient had both a subacute chronic deep venous thrombosis (DVT) in the right arm vessels and nephrotic range proteinuria. As is well known, proteinuria is a condition that causes thrombosis tendency and leads to the development of thromboembolism [4]. Despite all these, we evaluated it as a PE secondary to pulmonary vasculitis associated with BD. The reasons for these are (1) The patient's high levels of acute phase proteins, albeit non-specific; (2) During pulmonary vasculitis, de novo (in situ) thrombosis, and infarcts can be seen secondary to inflammation in the vessel wall. (3) Thromboembolism is not expected from DVTs in the course of BD, because the clot is tightly attached to the vessel wall due to inflammation in the vessel wall [5]. However, thromboembolism has rarely been reported in patients with intracardiac thrombosis (IC) [6]. The absence of IC thrombus in the echocardiography (ECHO) examination in our patient excludes this possibility. Hemoptysis and alveolar bleeding are seen in patients with PE, as in PAA. Pulmonary vasculitis and thrombosis of pulmonary vessels may cause infarctions, focal or diffuse hemorrhages, and focal areas of atelectasis [7]. Interestingly, PAA may develop later in vessels with PE. Seyahi et al. observed the development of PAA over time in some of the patients with PE. Hence, they stated that PEs might be a forerunner for PAA [3]. In this respect, diagnosis of PE as thromboembolism due to causes other than BD and administration of anticoagulants will both delay the treatment of immunosuppressive and increase mortality by creating a serious risk of bleeding. Systemic anti-inflammatory therapy is the mainstay for PE and PAA in the course of BD. Recent studies reported improved outcomes with the use of immunosuppressants and high-dose steroids in all types of vascular involvements [8].

## Conclusions

Even if a diagnosis of PE according to CT angiography chest is reported in patients with BD with hemoptysis and the patient has risk factors for thromboembolism, first of all, pulmonary vasculitis associated with BD should be considered, at least this possibility should be excluded first. Early initiation of high-dose steroid and immunosuppressive drugs will positively affect the outcome of the disease. This case highlights the importance of having a high clinical suspicion and knowledge of vascular involvement in BD patients presenting with new onset of fever, shortness of breath, hemoptysis.
